# Malaria Vectors in Lake Victoria and Adjacent Habitats in Western Kenya

**DOI:** 10.1371/journal.pone.0032725

**Published:** 2012-03-08

**Authors:** Noboru Minakawa, Gabriel O. Dida, George O. Sonye, Kyoko Futami, Sammy M. Njenga

**Affiliations:** 1 Department of Vector Ecology and Environment, Institute of Tropical Medicine (NEKKEN) and Global Center of Excellence Program, Nagasaki University, Nagasaki, Japan; 2 School of Public Health, Maseno University, Maseno, Kenya; 3 Springs of Hope, Mbita, Kenya; 4 Eastern and Southern Africa Centre of International Parasite Control, Kenya Medical Research Institute, Nairobi, Kenya; Instituto de Higiene e Medicina Tropical, Portugal

## Abstract

The prevalence of malaria among the residents of the Lake Victoria basin remains high. The environment associated with the lake may maintain a high number of malaria vectors. Lake habitats including water hyacinths have been suspected to be the source of vectors. This study investigated whether malaria vectors breed in the lake habitats and adjacent backwater pools. Anopheline larvae were collected within the littoral zone of the lake and adjacent pools located along approximately 24.3 km of the lakeshore in western Kenya, and their breeding sites characterized. Three primary vector species, *Anopheles arabiensis*, *Anopheles gambiae* s.s. and *Anopheles funestus* s.s., and three potential vectors, were found in the lake habitats. Unexpectedly, *An. arabiensis* was the most dominant vector species in the lake sampling sites. Its habitats were uncovered or covered with short grass. A potential secondary malaria vector, *Anopheles rivulorum*, dominated the water hyacinths in the lake. Most breeding sites in the lake were limited to areas that were surrounded by tall emergent plants, including trees, and those not exposed to waves. Nearly half of adjacent habitats were lagoons that were separated from the lake by sand bars. Lagoons contained a variety of microhabitats. *Anopheles arabiensis* dominated open habitats, whereas *An. funestus* s.s. was found mainly in vegetated habitats in lagoons. The current study confirmed that several breeding sites are associated with Lake Victoria. Given that Lake Victoria is the second largest lake in the world, the lake related habitats must be extensive; therefore, making targeted vector control difficult. Further exploration is necessary to estimate the effects of lake associated habitats on malaria transmission so as to inform a rational decision-making process for vector control.

## Introduction

While malaria transmission has been reduced considerably in some Kenyan regions, it still remains high among the residents in the Lake Victoria basin [1], [2]. Intense malaria transmission is often observed in villages near lakes and large reservoirs in Africa [3], [4]. Studies have shown that malaria cases increase with decreasing distance to the shores of large water bodies [5], [6]. Construction of dams may shift seasonal transmission to perennial transmission [7]. Thus, it is reasonable to hypothesize that the environment associated with the shoreline of Lake Victoria supports a high density of malaria vectors given the year-round availability of water.

The major African malaria vectors, *Anopheles arabiensis* and *Anopheles gambiae* s.s., often inhabit small and sunlit temporary water pools [8]–[10], Numerous small water pools in the wetlands along the lakeshore may become suitable vector habitats as has been demonstrated from the presence of malaria vectors in stagnant water pools on the lakeshore where tall vegetation has been cleared [11]. Although recent studies suggest that breeding occurs in permanent and semi-permanent water pools [12], [13], the characteristics of their habitats suggest that they do not breed within the lake water itself [14].

Another important malaria vector, *Anopheles funestus* s.s., usually inhabits large, stable water pools covered with aquatic vegetation that can be found in wetlands adjacent to Lake Victoria [8], [9], [15]. These habitats may exist throughout the year because of a constant supply of seepage water from the lake. Malaria vectors also inhabit large back-water pools (lagoons) along the shore of Lake Victoria [16]. Lagoons appear on strips of land that emerge following reductions in the lake water levels. Waves transport sands, which build up along the shore. The body of water that becomes enclosed behind the mounds of sand creates a lagoon. If a lagoon is large and stable, emergent and floating plants colonise the area. Previously, three major vector species have been recorded in such sites, although the micro-habitats of these vectors within lagoons have not been documented in detail [16].

Given that *An. funestus s.s.* is closely associated with aquatic vegetation [8], [9], [15], it has been proposed that the infestation of water hyacinths (*Eichhornia crassipes*) in African lakes has increased breeding site availability for this vector species [17], [18]. A potential secondary vector, *Anopheles rivulorum*, may also occur in water hyacinths, because the larvae of this species are often associated with floating plants such as Nile cabbage (*Pistia stratiotes*) [19]. The water hyacinths originated from South America, and were first reported in Lake Victoria in 1989. Since then, they have quickly spread throughout the lake, covering 80% of the Ugandan coastline by 1995 [20]. One study found no malaria vectors amongst water hyacinth supported sites in Lake Victoria [17], whilst another reported larvae of the *An. funestus* complex within a water hyacinth mat at the margin of the lake [18].

Lake Victoria is the second largest lake in the world, and the effects of the lake habitats on local malaria transmission could be substantial if they support vector breeding. This threat would not be limited to Lake Victoria, as water hyacinths have already invaded several lakes and reservoirs in malaria endemic areas of Africa [21]. Here, we investigated whether the habitats associated with Lake Victoria serve as active breeding sites for malaria vectors.

## Materials and Methods

### Study area

**Figure 1:**
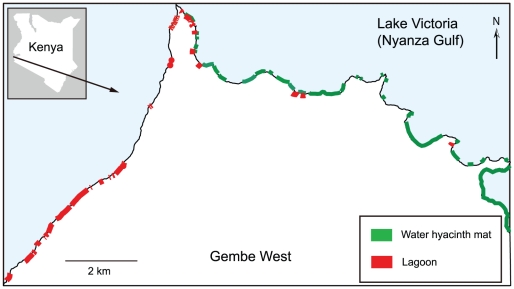
Distribution of water hyacinth mats and lagoons.

The study area included the entire shoreline of Lake Victoria in the Gembe West region of Mbita District, western Kenya (Figure 1). The area forms a peninsula surrounded by lake water to the north, west, and east. The eastern shore is within the Nyanza Gulf, and the western shore faces the main lake body. The total shoreline of the study area was approximately 24.3 km, with the eastern and western shores comprising 16.2 km and 8.1 km, respectively. The western shore faces the main lake body and is exposed to strong waves generated by westerly winds. Strong winds from the main lake body are common in this area in the afternoon. Sands carried by waves accumulate on the western shore, and several lagoons have formed behind the sand mounds. While the wind creates strong waves on the western side, the shadow effect of the peninsula creates a calm area along the eastern shore. Most residents depend on fishing and traditional small-scale farming. Two wet seasons occur annually from March to June and October to November, but the periods vary each year. A high prevalence of malaria is known among the local residents (≥40% *Plasmodium falciparum* parasite rate in the overall population) [2]. Three species of malaria vectors were previously identified: *An. arabiensis*, *An. gambiae* s.s., and *An. funestus* s.s. [18], [22].

### Water hyacinth habitats in the lake

The distribution of water hyacinths was investigated along the lakeshore using a small engine boat in March 2008. The water hyacinth mats that were drifting across the lake were not surveyed. When a water hyacinth mat was observed along the shore, its location was recorded using a hand-held global positioning system (GPS; GPSmap 60CSx, Garmin International, Inc., Olathe, Kansas, USA). The scattered water hyacinths were not considered to constitute a mat; only the water hyacinths that were densely assembled into a mat were assessed. When tall emergent vegetation blocked the view from the boat, the presence of water hyacinths was confirmed from the land. The lengths of short water hyacinth mats were estimated using a laser rangefinder (Nikon Laser 550AS; Nikon Vision Co. Ltd., Tokyo, Japan), while those of longer mats were estimated using a geographical information system (GIS; Arc View 9.2; Environmental Systems Research Institute, Redlands, California, USA). Both ends of each mat were located using the GPS and their locations were plotted on the map produced by a satellite image that was taken in June 2006 (0.6-m ground resolution; Quick Bird image; Digital Globe, Longmont, Colorado, USA).

### Larval sampling

Each water hyacinth mat in the lake was examined for anopheline larvae using a standard mosquito dipper (350 ml; BioQuip Products, Rancho Dominguez, California, USA) in June 2008 (during the wet season). When a mat was longer than 100 m, it was divided into 100-m sections using the GIS. Each site was dipped a total of 100 times, and a density was estimated as a total number of larvae found in 100 dips per site The same sampling procedure was repeated for the same sites in February 2009 (during the dry season), and also applied to the other habitats described below. The sampling sites were characterized based on the presence of surrounding trees or non-woody tall emergent plants as these conditions may influence the occurrence of malaria vectors. Water hyacinths were often found trapped by rows of trees (*Aeschynomene elaphroxylon*) that were planted in the lake water to discourage hippopotami (*Hippopotamus amphibious*) from approaching farms near the lake [17], [18].

### Non-water hyacinth habitats in the lake

During the dry season, mosquitoes were also sampled from various types of non-water hyacinth habitats in the lake. These habitats were only surveyed during this period, because they were not easily accessible due to dense vegetation during the wet season. Such habitats were within the vicinity of (i.e., could be seen from) the water hyacinth sampling sites. The non-water hyacinth habitats were categorized based on the types of vegetation cover: (1) tall vegetation (greater than approximately 1 m), (2) short vegetation including railing plants (less than approximately 1 m), and (3) minimal vegetation (uncovered). Tall vegetation included papyrus and sedges, but not trees. The uncovered habitat was a distinct open area larger than approximately 1 m^2^. All uncovered areas were surrounded by vegetation and not directly exposed to waves. Dipping sites were limited within approximately 5 m of the land.

### Habitats adjacent to the lake

All lagoons in the study area were located in February 2009, and their geographical positions were recorded using a GPS. The stagnant water pools that were not associated with a sand bar were also located along the lakeshore. The surveyed water pools were categorised based on their composition: (1) lagoons, (2) other natural pools that were not separated by sand bars from the lake, and (3) man-made pools such as ditches and ponds that were dug to draw water from the lake for farming. The survey did not include temporary water pools such as animal hoof prints or small puddles because these habitats mostly occur on the fringes of larger stagnant pools. Despite the large number of these temporary pools, their importance in malaria transmission is expected to be low [10]. The survey was limited to the strip of land (within approximately 100 m at most from the lake) that had emerged along the shore after a recent reduction in the lake water [16].

When a water pool contained multiple distinct microhabitats, the habitats were categorised based on the types of main vegetation cover: (1) tall vegetation, (2) short vegetation including trailing plants, (3) floating plants, and (4) uncovered. Anophelines were sampled from each microhabitat.

### Species identification

The sampled larvae were immediately preserved in 96% ethanol at the field site and identified microscopically in the lab using a morphological identification key [23]. The specimens belonging to the *An. funestus* and the *An. gambiae* complexes were further identified by species using the ribosomal DNA-polymerase chain reaction (PCR) method [24]. The specimens that were microscopically indistinguishable were also examined using PCR. The primers specific to *An. funestus* s.s., *An rivulorum*, *Anopheles leesoni*, *Anopheles parensis*, *An. Arabiensis*, and *An. gambiae* s.s. were used for the PCR.

### Data analyses

A generalised linear model (GLM; the ‘stat’ and ‘MASS’ packages in R version 2.13.1) was used to determine whether the surrounding vegetation type (tree, non-woody plant or short plant) influence each anopheline species in the water hyacinths. Data sets from two sampling years were included in the initial model to assess the temporal effect and fix the effect; sampling year was included as an explanatory variable. Since the response variable (numbers of larvae) was counts, a poisson regression was applied to optimize the statistical model. A negative binomial regression was applied when data were overdispersed. When a data set included excess zeroes (absence of larvae), a zero-inflated regression model and a hurdle model (the ‘pscl’ package in R) were also applied. Both models are two-component mixture models combining a count regression part and a binomial regression part [25]. An optimal statistical model was selected using the Akaike Information Criteria (AIC). The significance of each explanatory variable remaining in the optimal model was estimated using a log-likelihood ratio test.

Using the same statistical procedure, the mosquito data set from water hyacinth habitats and non-water hyacinth habitats in the lake in 2009 was analyzed together, to determine whether surrounding vegetation type and microhabitat type based on vegetation cover (water hyacinth, covered with short vegetation, covered with tall vegetation, and uncovered) affect mosquitoes. Similarly, the effects of water pool type (lagoon, other natural pool, and man-made pool) on mosquitoes were estimated. Including both data sets from the lake and adjacent pools, the effects of microhabitat and macrohabitat (lake and pool) were also estimated. The dominant vector species in the lake habitats and adjacent pools were also determined using the same statistical methods. In this case, the response variable was the number of larvae, and the explanatory variable was the species.

In addition to the univariate analysis for each species, a redundancy analysis (RDA: the ‘vegan’ package in R) was also applied to the data. This multivariate method comprehensively analyses the relationships between various species and the explanatory variables. An RDA is more useful for a data set including many zeroes than is a canonical correspondence analysis because it is less sensitive to double zeroes [26]. A square root transformation was applied to the raw species data to down-weigh the effect of abundant species. Subsequently, the chord transformation was applied to minimise the double-zero problem. The significance of all explanatory variables together was assessed using permutation tests. Additionally, each explanatory variable was tested, and the optimal model was then selected by excluding variables that were not significant. The significance of each axis was also assessed using permutation tests.

### Ethics statement

This study was approved by the Scientific Steering Committee and National Ethics Review Committee of the Kenya Medical Research Institute (SSC No. 1310), and the ethics review committee of Nagasaki University. No specific permits were required for the described field studies. The study area did not include a national park or other protected area of land. However, we obtained consents from land owners when we collected mosquitoes on privately-owned lands. This field study did not involve endangered or protected species.

## Results

### Water hyacinth habitats in the lake

A total of 43 distinct water hyacinth mats were found along the lakeshore in 2008 (Figure 1). All mats were on the eastern shore. The lengths of the mats ranged from 27.2 to 1585.2 m (mean ± SE; 184.2±18.2 m). The total length of the mats was 7.9 km. Water hyacinths covered one third (33.8%) of the total shoreline surveyed and nearly half (48.8%) of the eastern shore.

In 2008, larvae were sampled from 93 water hyacinth sites; however, this decreased to 58 owing to a reduction in water hyacinths in 2009. Of the 151 sites, 36 (23.8%) contained water hyacinths that were trapped or surrounded by trees. In the first survey, 797anopheline larvae were collected from 69 sites, while 508 larvae were collected at 29 sites in the second survey. A total of 1047 larvae were identified by species or cryptic species complex. Three primary malaria vectors, *An. funestus* s.s., *An. arabiensis* and *An. gambiae* s.s., were recorded from the water hyacinths (Table 1). However, *An. arabiensis* and *An. gambiae s.s.* were excluded from the analysis, as their numbers were insufficient for determining the dominant species in water hyacinth habitats. In addition, *Anopheles rivulorum*, *Anopheles lessoni*, *Anopheles pharoensis* and *Anopheles coustani* s.l. were recorded from the water hyacinths. Since *P. falciparum* were found in *An. rivulorum* in Tanzania, this species was also included in the statistical analysis [27], [28]. Although *P. falciparum* were reported from *An. lessoni* and *An. pharoensis* [29], [30], their numbers were insufficient for the statistical analysis. *Anopheles coustani* s.l. was also excluded from the analysis, because the sample might include multiple species, and the species group is considered unimportant for transmission [31].

**Table 1:** Mean densities ± SE (per site) and occurrences of anopheline larvae sampled from the water hyacinths that were surrounded by trees or other tall emergent plants, or that were exposed to waves in the lake in 2008 and 2009.

Taxa	Trees (n = 36)	Non-woody tall plants (n = 71)	Short plants (exposed) (n = 44)	2008 (n = 93)	2009 (n = 58)	Total (n = 151)	No. of larvae (%) (n = 1047)
*An. funestus* s.s.*	2.4±0.8 (13)^a^	0.0±0.0 (0)^b^	0.1±0.1 (2)^b^	0.8±0.3 (11)	0.3±0.2 (4)	0.6±0.1 (15)	91 (8.7)
*An. lessoni*	0.1±0.1 (3)	0 (0)	0 (0)	<0.1 (2)	<0.1 (1)	<0.1 (3)	3 (0.3)
*An. rivulorum* *	3.5±1.1^a^ (14)	0.8±0.5^b^ (12)	0.3±0.2^b^ (4)	1.3±0.4 (22)	1.3±0.7 (8)	1.3±0.4 (30)	195 (18.6)
*An. arabiensis*	0.7±0.6 (4)	0.1±0.0 (3)	0.2±0.2 (2)	0.1±0.0 (4)	0.6±0.4 (5)	0.3±0.1 (9)	38 (3.6)
*An. gambiae* s.s.	0 (0)	0 (0)	0.1±0.1 (2)	<0.1 (1)	<0.1 (1)	<0.1 (2)	3 (0.3)
*An. pharoensis*	0 (0)	0.2±0.2 (3)	0.1±0.0 (2)	0.1±0.1 (1)	0.3±0.2 (4)	0.1±0.1 (5)	20 (1.9)
*An. coustani* s.l.	6.2±1.4 (24)	3.8±0.8 (38)	4.6±1.4 (18)	5.1±0.8 (59)	3.9±1.2 (20)	4.6±0.6 (79)	697 (66.6)

The numbers in parentheses after the density value indicate positive sites. The values with the same letter were not statistically significant (P>0.05). Where letters are missing on densities, the count portion was excluded from the optimal zero-inflated regression model or hurdle model. Similarly, the binomial portion was excluded where letters are missing on the numbers of positive sites.

* Species included in the analyses.

A hurdle model with a negative binomial distribution had the lowest AIC, and the optimal model included the binomial portion only. *Anopheles rivulorum* occurred in the water hyacinth mats more frequently than *An. funestus* s.s. (P = 0.017). When *An. rivulorum* was analyzed separately to reveal the important vegetation types that trapped the water hyacinths, a GLM with negative binomial distribution revealed that the vegetation variable was statistically significant (P = 0.007). A multiple comparison test revealed that the density of the species in water hyacinths trapped by trees was significantly higher than under other conditions (Table 1). For *An. funestus* s.s., the vegetation variable was only significant in the binomial portion of the hurdle model (P<0.001), and its occurrence in the water hyacinths trapped by trees was significantly greater. The sampling year was not statistically significant for both species.

**Figure 2:**
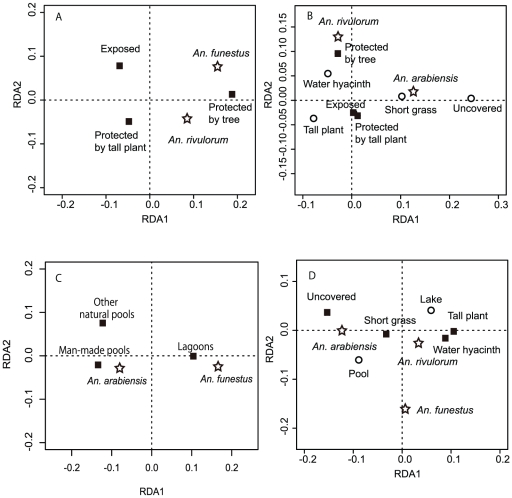
Redundancy analyses results showing the relationship between the vector species and the environmental factors for each habitat type: (A) hyacinth habitats, (B) lake habitats, (C) adjacent habitats, and (D) lake and adjacent habitats. Only the significant explanatory variables are included in the plots. The first axis was significant in the biplot for all cases, but the second axis was not significant in the analysis with water hyacinths.

An RDA also indicated that the vegetation type that trapped water hyacinths was a significant variable (P<0.001), but the sampling year was not. The biplot indicated that *An. funestus* s.s. had a greater association with the water hyacinths trapped by trees than *An. rivulorum* (Figure 2A).

### All lake habitats

Together with water hyacinth habitats, a total of 233 sites were dipped in the littoral zone in 2009. A total of 1,243 anophelines were collected at 112 sites, and 1,047 larvae were identified (Table 2). Although all three primary vector species were recorded, nearly 90% of the identified larvae were *An. arabiensis*. Only *An. arabiensis* and *An. rivulorum* were statistically analyzed. The numbers of the other species were insufficient for the analysis. A negative bionomial hurdle model showed the lowest AIC when the abundance was compared between *An. arabiensis* and *An. rivulorum*. The count portion was excluded from the optimal model, and the result from the binomial portion indicated that the occurrence of *An. arabiensis* was significantly greater than that of *An. rivulorum* in the lake habitats (P = 0.002). When the abundance of *An. arabiensis* was regressed against the microhabitat categorized by vegetation cover and the vegetation type that surrounded the sampling sites, the microhabitat variable remained in the binomial portion of the hurdle model, while the surrounding vegetation variable remained in the count portion. The occurrence of *An. arabiensis* was significantly greater in short grass and uncovered habitats compared with the habitats containing tall vegetation (P<0.001). The density of *An. arabiensis* was significantly greater in the habitats surrounded by non-woody tall plants than those exposed to waves (P = 0.022). When *An. rivulorum* was regressed against these variables, a negative binomial hurdle model revealed that both variables were significant (P = 0.012 for microhabitat, and P<0.001 for surrounding vegetation type). The occurrence of this species was significantly greater in the water hyacinths located in the habitats surrounded by trees (P<0.001). The multivariate analysis also indicated that the vegetation surrounding the habitats and the microhabitat were significant variables (P<0.015 and P<0.001, respectively) (Figure 2B). The biplot indicated that *An. arabiensis* was positively associated with short grass or uncovered habitats, while *An. rivulorum* was positively associated with water hyacinths surrounded by trees.

**Table 2:** Mean densities ± SE (per site) and occurrences of anopheline larvae sampled from the water hyacinth and non-water hyacinth habitats that were surrounded by trees or other tall emergent plants or were exposed to waves in the lake, and from microhabitats categorized based on vegetation cover at each sampling site in 2009.

	Surrounded by		Microhabitats					Total	
Taxa	Trees (n = 55)	Non-woody tall plants (n = 146)	Short plants (exposed) (n = 32)	Water hyacinth (n = 58)	Short (n = 64)	Tall (n = 94)	Uncovered (n = 17)	Habitats (n = 233)	No. of larvae (%) (n = 1047)
*An. funestus s.s.*	0.3±0.2 (3)	0 (0)	0.1±0.1 (1)	0.3±0.2 (4)	0 (0)	0 (0)	0 (0)	0.1±0.1 (4)	18 (1.7)
*An. lessoni*	<0.1 (1)	0 (0)	0 (0)	<0.1 (1)	0 (0)	0 (0)	0 (0)	<0.1 (1)	1 (0.1)
*An. rivulorum* *	1.3±0.7 (10)^a^	0.1±0.1 (4)^b^	0.3±0.3 (1)^b^	1.3±0.6 (8)^a^	0.1±0.1 (3)^b^	0.1±0.1 (4)^b^	0.0±0.0 (0)^b^	0.4±0.3 (15)	101 (9.6)
*An. arabiensis* *	0.8±0.4^ab^ (8)	1.7±0.5^a^ (24)	0.5±0.3^b^ (5)	0.8±0.4 (6)^a^	2.4±1.1 (20)^b^	<0.1 (2)^a^	5.4±1.6 (9)^b^	1.2±0.7 (37)	283 (27.0)
*An. gambiae s.s.*	0 (0)	0.1±0.1 (4)	0.1±0.1 (1)	<0.1 (1)	0.2±0.1 (3)	0 (0)	0.1±0.1 (1)	0.1±0.1 (5)	15 (1.4)
*An. pharoensis*	0 (0)	0.3±0.1 (10)	0.1±0.0 (2)	0.3±0.2 (4)	0.1±0.1 (3)	0.1±0.0 (3)	0.7±0.5 (2)	0.2±0.1 (12)	41 (3.9)
*An. maculipalpis*	<0.1 (1)	0 (0)	0 (0)	0 (0)	0 (0)	0 (0)	<0.1 (1)	<0.1 (1)	2 (0.2)
*An. coustani* s.l.	6.4±1.2 (37)	0.9±0.2 (28)	3.4±1.8 (7)	3.9±1.2 (20)	2.0±0.6 (20)	2.6±0.7 (32)	0 (0)	2.5±0.9 (72)	586 (56.0)

The number in parentheses below the density value indicates positive sites. The values with the same letter were not statistically significant (P>0.05). Where letters are missing on densities, the count portion was excluded from the optimal zero-inflated regression model or hurdle model. Similarly, the binomial portion was excluded where letters are missing on the numbers of positive sites.

* Species included in the analyses.

### Habitats adjacent to the lake

A total of 95 distinct stagnant pools were identified along the lakeshore. Of these, 43 (45.3%) pools were lagoons, of which 34 (79.1%) were located along the western shore. The study area also included 44 (46.3%) man-made pools and eight (8.4%) other natural pools. The lengths of the lagoons ranged from 4.5 to 652 m (mean ± SE; 135.3±18.2 m). The total length of all lagoons was 6.0 km, with 4.6 km belonging to the western shore (56.8%), (Figure 1). Tall plants and floating plants were more strongly associated with lagoons. All floating-plant habitats included only water hyacinths, with the exception of one habitat that included both the water hyacinth and Nile cabbage.

A total of 149 microhabitats within stagnant pools were sampled for mosquitoes in 2009 (Table 3). Most lagoons harboured multiple microhabitats. Because of the strong association between the water-pool type and the microhabitat, the association of vector species was analyzed separately for these two variables. A total of 5,934 anopheline larvae were collected from 117 sites. A total of 5,673 larvae were identified, of which 5,085 (89.6%)were *An. arabiensis*.

**Table 3:** Numbers of microhabitats sampled in the three types of water pools that were adjacent to the lake.

Water-pool type	Water hyacinth	Short	Tall	Open	Total
Lagoons	9	30	20	24	83
Other natural pools	0	7	0	8	15
Man-made pools	0	11	0	40	51
Total	9	48	20	72	149

GLMs with negative binomial distribution had the lowest values of AIC for explaining the relationships between mosquito abundance and water-pool type. The statistical analysis was only applied to *An. funestus* s.s. and *An. arabiensis*, because the numbers of the other species were insufficient. The optimal model included the variable of water-pool type for both species (P<0.001 for both cases). The multiple comparison test indicated that the density of *An. funestus* s.s. in lagoons was significantly higher than that in the other pool types, whereas the density of *An. arabiensis* in man-made pools was significantly higher (Table 4). An RDA also revealed a significant effect of pool habitat type (P<0.001) (Figure 2C). *Anopheles arabiensis* was positively correlated with man-made and other natural pools, whereas *An. funestus* s.s. was positively associated with lagoons.

**Table 4:** Mean density ± SE (per site) and occurrences of anopheline larvae sampled from each type of water pool in 2009.

Taxa	Lagoons (*n* = 83)	Other natural pools (*n* = 15)	Man-made pools (*n* = 51)	Total (*n* = 149)	No. of larvae (%) (n = 5673)
*An. funestus s.s.* *	2.4±0.7^a^ (25)	0^b^ (0)	0.2±0.2^b^ (1)	1.4±0.4 (26)	204 (3.6)
*An. lessoni*	<0.1 (2)	0 (0)	0 (0)	<0.1 (2)	2 (<0.1)
*An. rivulorum*	0.5±0.2 (8)	0 (0)	0.1±0.1 (1)	0.3±0.1 (9)	44 (0.8)
*An. arabiensis* *	14.9±6.8^a^ (34)	13.3±7.0^a^ (8)	71.6±19.2^b^ (29)	34.1±7.9 (71)	5085 (89.6)
*An. gambiae s.s.*	0.4±0.3 (6)	1.3±1.3 (1)	0.2±0.2 (1)	0.4±0.2 (8)	61 (1.1)
*An. pharoensis*	0 (0)	1.9±1.8 (2)	<0.1 (1)	0.2±0.2 (3)	30 (0.5)
*An. coustani* s.l.	2.1±0.6 (27)	1.2±0.6 (4)	1.1±0.5 (8)	1.7±0.4 (39)	261 (4.6)

The number in parentheses after the density value indicates positive sites. Densities with the same letter did not significantly differ. Where letters are missing on densities, the count portion was excluded from the optimal zero-inflated regression model or hurdle model. Similarly, the binomial portion was excluded where letters are missing on the numbers of positive sites.

* Species included in the analyses.

### All habitats associated with the lake

When the data sets from the lake and the land habitats in 2009 were combined, a total of 7177 anopheline larvae were recorded from 229 of 382 sites, of which 6720 were identified (Table 5). Nearly 80% of the larvae were *An. arabiensis* followed by *An. funestus* s.s. and *An. rivulorum*. The numbers of the other species identified were insufficient for statistical analyses. A GLM with a negative binomial distribution revealed that the density of *An. arabiensis* was significantly higher than that of other species (P<0.001). A GLM with a negative binomial distribution revealed that the densities of *An. arabiensis* and *An. funestus* s.s. were significantly lower in the lake habitats than the land habitats (P<0.001 for both species); however, this difference was not significant for *An. rivulorum*. The microhabitat variable was significant for all cases (P<0.001). The densities of *An. funestus* s.s. and *An. rivulorum* were significantly higher in the water hyacinths. The density of *An. arabiensis* was significantly higher in uncovered habitat.

**Table 5:** Mean densities ± SE (per site) and occurrences of anopheline larvae sampled from the lake and adjacent habitats, and from microhabitats categorized based on vegetation cover at each sampling site in 2009.

	Macrohabitats		Microhabitats				Total	
Taxa	Lake (n = 233)	Pool (n = 149)	Water hyacinth (n = 68)	Short (n = 112)	Tall (n = 113)	Uncovered (n = 89)	Habitats (n = 382)	No. of larvae (%) (n = 6720)
*An. funestus s.s.* *	0.1±0.0 (4)	1.4±0.4 (26)	1.2±0.7^a^ (8)	0.7±0.3^b^ (10)	0.5±0.2^b^ (10)	<0.1^c^ (2)	0.6±0.2^A^ (30)	222 (3.3)
*An. lessoni*	<0.1 (1)	<0.1 (2)	<0.1 (1)	<0.1 (1)	<0.1 (1)	0 (0)	<0.1 (3)	3 (<0.1)
*An. rivulorum* *	0.4±0.2 (15)	0.3±0.1 (9)	1.5±0.6^a^ (11)	0.1±0.1^b^ (6)	0.2±0.1^b^ (6)	0.1±0.1^b^ (1)	0.4±0.1^A^ (24)	145 (2.2)
*An. arabiensis* *	1.2±0.3 (37)	34.1±7.9 (71)	1.4±0.8^a^ (8)^a^	6.4±2.1^a^ (40)^b^	0.2±0.1^b^ (52)^b^	51.0±12.7^c^ (52)^b^	14.1±3.2^B^ (108)	5368 (79.9)
*An. gambiae s.s.*	0.1±0.0 (5)	0.4±0.2 (8)	0.1±0.1 (2)	0.1±0.1 (5)	0 (0)	0.6±0.3 (6)	0.2±0.1 (13)	76 (1.1)
*An. pharoensis*	0.2±0.1 (12)	0.2±0.2 (3)	0.2±0.2 (4)	0.1±0.1 (4)	0.1±0.0 (3)	0.5±0.3 (4)	0.2±0.1 (15)	71 (1.1)
*An. maculipalpis*	<0.1 (1)	0 (0)	0 (0)	0 (0)	0 (0)	<0.1 (1)	<0.1 (1)	2 (<0.1)
*An. coustani* s.l.	2.5±0.4 (72)	1.7±0.4 (40)	3.5±1.0 (22)	2.3±0.5 (41)	2.7±0.6 (44)	0.3±0.2 (5)	2.2±0.3 (112)	833 (12.4)

The number in parentheses after the density value indicates positive sites. The values with the same letter were not statistically significant (P>0.05). Where letters are missing on densities, the count portion was excluded from the optimal zero-inflated regression model or hurdle model. Similarly, the binomial portion was excluded where letters are missing on the numbers of positive sites.

* Species included in the analyses.

The multivariate analysis also revealed that the variables of lake or land, as well as the microhabitat were significant (P<0.001 and P<0.001, respectively) (Figure 2D). The biplot indicated that *An. rivulorum* was positively associated with water hyacinths, tall plants, and lake, while *An. arabiensis* was associated with short grass, uncovered habitat, and land habitat. The association of *An. funestus* s.s. with the micro- and macro-habitats was not as clear as for the other species.

## Discussion

### Lake habitats

This study confirms that African malaria vectors breed in Lake Victoria. Surprisingly, *An. arabiensis* was the most dominant vector species in the lake sampling sites, contrary to previous studies which reported this vector to mainly breed in small and sunlit temporary pools [8]–[10]. This species was mostly found in the lake areas covered with short grass, as well as uncovered areas. However, *An. arabiensis* was less common in the areas that were exposed to waves. Tall emergent plants surrounding the habitats must create an isolated and sheltered environment similar to breeding sites on land. The number of positive sites and their density suggests that the occurrence of this species in the lake was extensive. While larvae may accidentally enter the lake from adjacent pools on the land during heavy rainfall [32], the possibility of oviposition in the lake habitats should not be excluded. On the other hand, the lake habitats may serve as reservoirs; migration from the lake habitats may contribute to breeding in newly formed adjacent pools.

A small number of *An. gambiae* s.s. also occurred in the lake. This species is less common in this study area, which may have been caused by the increase in bed net coverage [33], [34]; therefore, low density and occurrence do not necessarily mean that this species is less adapted to the lake habitats as compared with *An. arabiensis*. Both species belong to the same species complex, and they are often sympatric [9], [22]. This is the first study to report both *An. arabiensis* and *An. gambiae* s.s. in a lake. Although they are the most important malaria vectors and have been studied extensively, few studies have explored their presence in lake habitats.

### Water hyacinth habitats

This study also confirmed that malaria vectors are able to breed amongst water hyacinth mats in Lake Victoria. Although larvae of the *An. funestus* complex were reported from a water hyacinth mat in the same study area [18], this is the first study to confirm the occurrence of malaria vectors within several water hyacinth mats in the lake. Other vector and potential vector species were also found in the water hyacinth mats in the lake; however, the results from the univariate and multivariate analyses suggest that, as compared with the other microhabitats in the lake, the water hyacinth habitat is more suitable for *An. rivulorum* than for the other species. *Anopheles funestus* s.s. was the second most abundant vector species in the water hyacinth mats in the lake. Both species are known to occur in vegetated habitats, and water hyacinths appear to be more suitable over other vegetation types [9], [15].

Although the results of the survey suggest that the lake is less suitable for *An. funestus* s.s., this species occurred in several water hyacinth mats trapped by a row of trees. *Anopheles rivulorum* was also more likely to occur in such habitats, but this tendency was greater for *An. funestus* s.s. than *An. rivulorum*. Conversely, fewer larvae were found in the water hyacinths trapped by non-woody emergent plants and in those exposed to waves. This suggests that the trees minimize the effects of waves and thereby stabilize the habitat. The water hyacinth habitat associated with trees must provide a similar environment to that of large vegetated pools on the land where this species usually occurs. Habitat stability is crucial for this mosquito species because their development takes approximately one month, which is, on average, double the period of *An. arabiensis* and *An. gambiae* s.s. [8], [35].

Dense water hyacinths covered nearly half of the eastern shore in this study area is located within the Nyanza Gulf. Since water movement is limited in this area, the water hyacinths often become trapped [17]. Trees are also common throughout the shoreline of the gulf, which trap the water hyacinths [17], [18]. Indeed, 22.4% of the water hyacinth sites surveyed in this study were associated with trees. This study also revealed that *An. rivulorum* could inhabit water hyacinth mats surrounded by tall non-woody emergent plants. Although its density was lower under these conditions, such habitats are more extensive than the tree habitats in the gulf.

### Habitats adjacent to the lake

Nearly half of stagnant water pools were lagoons, and most lagoons were located along the western shore and around the northern tip of the peninsula. The landscape and geomorphological features appear to largely determine where lagoons and water hyacinths occur. Most lagoons were large enough to host various types of vegetation, which, in turn, offer a variety of microhabitats. In contrast, the other types of natural and man-made pools had neither tall emergent plants nor floating plants. As a result, lagoons had greater densities of *An. funestus* s.s. and *An. rivulorum*. Lagoons also had several patches of open habitat in which *An. arabiensis* and *An. gambiae* s.s. mainly occurred. In fact, *An. arabiensis* was the most dominant species found in lagoons. Larvae of *An. arabiensis* likely occur at high densities if conditions are suitable, as this species requires less development time than *An. funestus* s.s. . However, to determine if lagoons produce more *An. arabiensis* compared with other vectors, the area covered by each microhabitat within lagoons must be estimated. If the area covered by vegetated habitats is larger than the area covered by open areas, the total production of *An. funestus* s.s. in lagoons may be higher than that of *An. arabiensis*.

Similarly, the production of malaria vectors in each pool type cannot be estimated using density alone. In fact, due to the high productivity of *An. arabiensis*, vector densities in man-made pools were much greater than those in lagoons and other natural pools. Although this study only measured the lengths of lagoons, the sizes of other pool types were obviously smaller than most lagoons. For example, most man-made pools were round, and their diameters were clearly less than five metres, whereas the widths of lagoons often exceeded five metres. The greater total length and number of lagoons imply that the total production of malaria vectors is greater for lagoons than for the other pool types. Moreover, the size of the lagoons indicates that they likely persist throughout the year, which would increase their total vector production [10]. Thus, lagoons likely play a more important role in local malaria transmission than do the other pool types along the shore of Lake Victoria. This hypothesis is further supported by the finding that lagoons are a major habitat of *An. funestus* s.s.. This species is more endophilic and anthropophilic than *An. arabiensis*; therefore, the former species is considered a more efficient vector [36]–[38].

Although this study did not examine inland water pools, previous studies have suggested that pools along the shore are more productive in the study area [12], [18]. Inland pools are mostly temporary, and they mainly produce vectors during the wet seasons [12], [18], [39], [40]. In contrast, constant seepage water from the lake stabilises the pools along the shore unless the lake water level fluctuates considerably [16]; therefore, pools along the shore are likely to produce malaria vectors throughout the year. Other possible vector sources include rivers and large reservoirs. However, this study area lacks permanent rivers with vegetated habitats and reservoirs. Small puddles may appear on dry beds of temporal streams, but most are temporary.

### Implications for vector control

The current study confirmed that several breeding sites are associated with Lake Victoria. Given that Lake Victoria is the second largest lake in the world, the habitats associated with the lake must be extensive, and their effects on local malaria transmission likely to be substantial. Although, further studies are necessary to estimate the effects of these habitats and the extent to which they contribute to local transmission, adjacent pool habitats require special attention.

Deployment of effective larval control operations targeting the habitats associated with the lake would be a challenge in this setting. Larviciding the habitats may not be practical given the limited residual effectiveness of current treatments in larger breeding sites. Reclaiming lagoons may be a temporary solution unless they are covered with a large amount of soil or concrete. Seepage water and waves will create numerous small breeding pools for *An. arabiensis* and *An. gambiae* s.s. on reclaimed land, though this could be avoided by planting trees [41]. Since such backwater habitats are important for nursing juvenile fish and other organisms [42], [43], reclamation requires additional care for environmental preservation. In fact, several juvenile fish were often observed in the lagoons during larval sampling. Moreover, new lagoons may appear after reclamation as the lake water level fluctuates [16]. A combination of locally suitable interventions is needed for effective control. Vector control may be enhanced by including the concept of integrated vector management through a rational decision-making process for the optimal use of multiple tools and resources in this study foci as well as similar areas throughout Sub-Saharan Africa [44], [45].

## References

[1] O'Meara WP, Bejon P, Mwangi TW, Okiro EA, Peshu N (2008). Effect of a fall in malaria transmission on morbidity and mortality in Kilifi, Kenya.. Lancet.

[2] Noor AM, Gething PW, Alegana VA, Patil AP, Hay SI (2009). The risks of malaria infection in Kenya in 2009.. BMC Infect Dis.

[3] Root GP (1999). Disease environments and subnational patterns of under-five mortality in sub-Saharan Africa.. Int J Popul Geogr.

[4] Keiser J, De Castro MC, Maltese MF, Bos R, Tanner M (2005). Effect of irrigation and large dams on the burden of malaria on a global and regional scale.. Am J Trop Med Hyg.

[5] Lautze J, McCartney M, Kirshen P, Olana D, Jayasinghe G (2007). Effect of a large dam on malaria risk: the Koka reservoir in Ethiopia.. Trop Med Int Health.

[6] Yewhalaw D, Legesse W, Van Bortel W, Gebre-Selassie S, Kloos H (2009). Malaria and water resource development: the case of Gilgel-Gibe hydroelectric dam in Ethiopia.. Malar J.

[7] Jobin W (1999). Dams and disease: Ecological design and health impacts of large dams, canals and irrigation systems.

[8] Gillies MT, DeMeillon B (1968). The Anophelinae of Africa, South of the Sahara (Ethiopian Zoogeographical Region).

[9] Gimnig JE, Ombok M, Kamau L, Hawley WA (2001). Characteristics of larval anopheline (Diptera: Culicidae) habitats in Western Kenya.. J Med Entomol.

[10] Minakawa N, Sonye G, Yan G (2005). Relationships between occurrence of Anopheles gambiae s.l. (Diptera: Culicidae) and size and stability of larval habitats.. J Med Entomol.

[11] Hancock GL (1934). The mosquitoes of namanve swamp, Uganda.. Journal of Animal Ecology.

[12] Fillinger U, Sonye G, Killeen GF, Knols BGJ, Becher N (2004). The practical importance of permanent and semipermanent habitats for controlling aquatic stages of Anopheles gambiae sensu lato mosquitoes: operational observation from a rural town in western Kenya.. Trop Med Int Health.

[13] Imbahale SS, Paaijmans KP, Mukabana WR, van Lammeren R, Githeko AK (2011). A longitudinal study on Anopheles mosquito larval abundance in distinct geographical and environmental settings in western Kenya.. Malaria J.

[14] Ndenga BA, Simbauni JA, Mbugi JP, Githeko AK, Fillinger U (2011). Productivity of malaria vectors from different habitat types in the western Kenya highlands.. PLoS ONE.

[15] Evans AM, Symes CB (1937). Anopheles funestus and its Allies in Kenya. 31.. Nature.

[16] Minakawa N, Sonye G, Dida GO, Futami K, Kaneko S (2008). Recent reduction in the water level of Lake Victoria has created more habitats for *Anopheles funestus*.. Malaria J.

[17] Ofulla AVO, Karanja D, Omondi R, Okurut T, Matano A (2010). Relative abundance of mosquitoes and snails associated with water hyacinth and hipp grass in the Nyanza gulf of Lake Victoria.. Lakes & Reservoirs: Research and Management.

[18] Minakawa N, Seda P, Yan G (2002). Influence of host and larval habitat distribution on the abundance of African malaria vectors in western Kenya.. Am J Trop Med Hyg.

[19] Lesson HS (1937). The mosquitoes of the *funestus* series in East Africa.. Bull Entomol Rse.

[20] Albright TP, Moorhouse TG, McNabb TJ (2004). The rise and fall of water hyacinth in Lake Victoria and the Kagera River Basin, 1989–2001.. Journal of Aquatic Plant Management.

[21] Navarro LA, Phiri G (2000). Water hyacinth in Africa: A survey of problems and solutions.

[22] Minakawa N, Mutero CM, Githure JI, Beier JC, Yan G (1999). Spatial distribution and habitat characterization of anopheline mosquito larvae in Western Kenya.. Am J Trop Med Hyg.

[23] Gillies MT, Coetzee M (1987). A supplement to the Anophelinae of Africa south of the Sahara (Afrotropical Region).

[24] Scott JA, Brogdon WG, Collins FH (1993). Identification of single specimens of the Anopheles gambiae complex by the polymerase chain reaction.. American Journal Of Tropical Medicine And Hygiene.

[25] Gurumu S, Trivedi PK (1996). Excess zeros in count models for recreational trips.. J Bus Econ Stat.

[26] Legendre P, Gallagher ED (2001). Ecologically meaningful transformations for ordination of species data.. Oecologia.

[27] Wilkes TJ, Matola YG, Charlwood JD (1996). *Anopheles rivulorum*, a vector of human malaria in Africa.. Med Vet Entomol.

[28] Temu EA, Minjas JN, Tuno N, Kawada H, Tkagi M (2007). Identification of four members of the *Anopheles funestus* (Diptera: Culicidae) group and their role in *Plasmodium falciparum* transmission in Bagamoyo coastal Tanzania.. Acta Trop.

[29] Carrara GC, Petrarca V, Niang M, Coluzzi M (1990). *Anopheles pharoensis* and transmission of *Plasmodium falciparum* in the Senegal River delta, West Africa.. Med Vet Entomol.

[30] Antonio-Nkondjo C, Kerah CH, Simard F, Awono-Ambene P, Chouaibou M (2006). Complexity of the malaria vectorial system in Cameroon: contribution of secondary vectors to malaria transmission.. J Med Entomol.

[31] Mbogo CN, Snow RW, Kabiru EW, Ouma JH, Githure JI (1993). Low-level Plasmodium falciparum transmission and the incidence of severe malaria infections on the Kenyan coast.. Am J Trop Med Hyg.

[32] Paaijmans KP, Wandago MO, Githeko AK, Takken W (2007). Unexpected high losses of Anopheles gambiae larvae due to rainfall.. PLoS ONE.

[33] Bayoh MN, Mathias DK, Odiere MR, Mutuku FM, Kamau L (2010). *Anopheles gambiae*: historical population decline associated with regional distribution of insecticide-treated bed nets in western Nyanza Province, Kenya.. Malaria J.

[34] Kawada H, Dida GO, Ohashi K, Komagata O, Kasai S (2011). Multimodal pyrethroid resistance in malaria vectors, *Anopheles gambiae* s.s., *Anopheles arabiensis*, and *Anopheles funestus* s.s. in Western Kenya.. PLoS ONE.

[35] Bayoh MN, Lindsay SW (2003). Effect of temperature on the development of the aquatic stages of Anopheles gambiae sensu stricto (Diptera: Culicidae).. Bull Entomol Res.

[36] Cohuet A, Simard F, Wondji CS, Antonio-Nkondjio C, Awono-Ambene P (2004). High malaria transmission intensity due to *Anopheles funestus* (Diptera: Culicidae) in a village of savannah–forest transition area in Cameroon.. J Med Entomol.

[37] Dia I, Ba H, Mohamed SA, Diallo D, Lo B (2009). Distribution, host preference and infection rates of malaria vectors in Mauritania.. Parasite Vector.

[38] Andrianaivolambo L, Domarle O, Randrianarivelojosia M, Ratovonjato J, Le Goff G (2010). Anthropophilic mosquitoes and malaria transmission in the eastern foothills of the central highlands of Madagascar.. Acta Trop.

[39] Minakawa N, Githure JI, Beier JC, Yan G (2001). Anopheline mosquito survival strategies during the dry period in western Kenya.. J Med Entomol.

[40] Mutuku FM, Bayoh MN, Gimnig JE, Vulule JM, Kamau L (2006). Pupal habitat productivity of Anopheles gambiae complex mosquitoes in a rural village in western Kenya.. Am J Trop Med Hyg.

[41] Hopkins GHE (1940). Afforestation as a method of drying up swamps.. E Afr Med J.

[42] Stephenson TD (1990). Fish reproductive utilization of coastal marshes of Lake Ontario near Tronto.. J Great Lakes Res.

[43] Minakawa N, Kraft GF (1999). Fall and winter diets of juvenile coho salmon in a small stream and an adjacent pond in Washington State.. J Fresh Ecol.

[44] WHO (2004). Global strategic framework for integrated vector management.

[45] Imbahale SS, Mweresa CK, Takken W, Mukabana WR (2011). Development of environmental tools for anopheline larval control.. Parsit Vectors.

